# Mesenteric rheumatoid nodules masquerading as an intra-abdominal malignancy: a case report and review of the literature

**DOI:** 10.1186/1477-7819-7-59

**Published:** 2009-07-15

**Authors:** Sumeer Thinda, James S Tomlinson

**Affiliations:** 1 VA Greater Los Angeles Healthcare System, Los Angeles, CA, USA; 2 Department of Surgery, David Geffen School of Medicine UCLA, Los Angeles, CA, USA

## Abstract

**Background:**

Rheumatoid nodules are the most common extra-articular findings in patients with rheumatoid arthritis. They occur most commonly at pressure points such as the extensor surfaces of the forearms, fingers, and occiput, but have also been reported to occur in unusual locations including the central nervous system, pericardium, pleura, and sclera. We present the unusual case of rheumatoid nodules in the small bowel mesentery masquerading as an intra-abdominal malignancy.

**Case presentation:**

A 65-year-old-male with a known history of longstanding erosive, nodular, seropositive rheumatoid arthritis was incidentally found to have a mesenteric mass on computed tomography (CT) exam of the abdomen. This mass had not been present on prior imaging studies and was worrisome for a malignancy. Attempts at noninvasive biopsy were nondiagnostic but consistent with a "spindle" cell neoplasm. Laparotomy revealed extensive thickening and fibrosis of the small bowel mesentery along with large, firm nodules throughout the mesentery. A limited bowel resection including a large, partially obstructing, nodule was performed. Pathology was consistent with an unusual presentation of rheumatoid nodules in the mesentery of the small bowel.

**Conclusion:**

Rheumatoid nodules should be considered in the differential diagnosis of a patient who presents with an intra-abdominal mass and a history of rheumatoid arthritis. Currently, no tests or imaging modality can discriminate with sufficient accuracy to rule out a malignancy in this difficult diagnostic delimma. Hopefully, this case will serve as impetus for further study and biomarker discovery to allow for improved diagnostic power.

## Background

**Figure 1:**
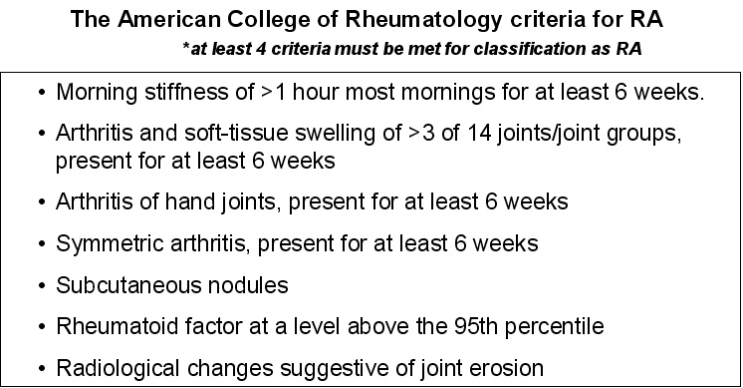
**Clinical criteria for the diagnosis of rheumatoid arthritis**. At least four of the seven criteria must be met for classification as RA.

Rheumatoid arthritis (RA) is a systemic inflammatory disease categorized as an autoimmune disorder, affecting about 1% of the United States population[1]. The pathophysiology is not completely understood but involves inappropriate activation of B and T cells which stimulates an inflammatory response most notably against synovial tissues of the body causing the classic chronic inflammatory arthritis[2]. This autoimmune disease is often associated with increased serum levels of Rheumatoid Factor (RF) which is an autoantibody against the constant region (Fc) of immunoglobulin G (IgG) type antibodies. The chronic inflammatory nature of this disease appears to be driven by cytokines, most notably by TNFa[2]. The diagnosis of RA is based on a spectrum of clinical criteria as listed in Figure 1[2]. Treatment of RA is founded in classic immunosuppressive therapy combined with newer agents targeting the specific inflammatory response of RA. An example of these newer agents is etanercept, which is a fusion protein combining the TNFa receptor with the Fc portion of the immunoglobulin protein. This molecule acts to dampen the effects of the excess TNFa released in patients with RA driving the inflammatory reaction [2].

In addition to the classic symptom of chronic inflammatory arthritis, RA is also associated with many extra-articular findings, including rheumatoid nodules, pyoderma gangrenosum, pericarditis, pleuritis, felty's syndrome, interstitial lung disease, glomerulonephritis, peripheral neuropathy, scleritis, episcleritis, and vasculitis[1,3]. Rheumatoid nodules are the most common extra-articular findings, occurring in about 25% of patients with RA[1]. Rheumatoid nodules occur most commonly at pressure points such as the extensor surfaces of the forearms, fingers, occiput, ischial areas, and the Achilles tendon[1]. They may also occur within internal tissues of the body: central nervous system, heart, pericardium, lungs, pleura, peritoneum, bones, vocal cords, and sclera[1]. Pulmonary nodules have been associated with pleural effusions, pneumothoraces, and fibrosis[4]. Cardiac nodules may be noted on echocardiogram and can cause symptoms of heart block and syncope[5,6]. There have been no reports of rheumatoid nodules within the mesentery, and as is exemplified by our case, this entity has the potential to masquerade as a malignancy.

## Case presentation

**Figure 2:**
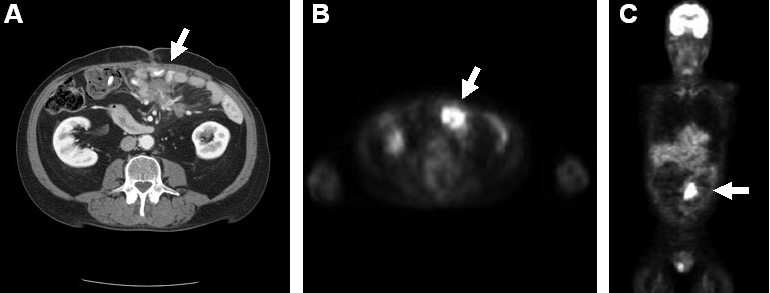
**Pre-operative CT and FDG-PET demonstrating the incidentally discovered mesenteric mass**. (A) The CT scan shows an ill-defined mass in the anterior aspect of the small bowel mesentery with linear adjacent fat stranding (arrow). (B) FDG-PET scan shows a heterogeneous moderately hypermetabolic focus in the anterior abdomen corresponding to the lesion on the CT scan (arrow). There are no other abnormal hypermetabolic foci. (C) Coronal image of the FDG-PET scan.

A 65-year-old-male with a known history of longstanding erosive, nodular, seropositive rheumatoid arthritis presented with the chief complaint of persistent fevers. His work up included chest and abdominal computed tomography (CT) which demonstrated both a pneumonia and an incidentally discovered mesenteric mass (Figure 2). After the pneumonia was treated and resolved, surgical oncology was consulted for further investigation of the mesenteric mass. Upon questioning, the patient admitted to intermittent crampy abdominal pain, occasional diarrhea, and a 5 lb unintentional weight loss over the previous 2 months. The patient's RA regimen consisted of etanercept and methotrexate prior to his pneumonia but these two immunosuppressive medications were withheld secondary to his diagnosis of pneumonia and workup for possible intra-abdominal malignancy. The patient's past surgical history was significant for sigmoidectomy for diverticulitis and a cholecystectomy for symptomatic cholelithiasis. Of note, there was no significant family history of malignancies. On examination, the patient was noted to have a well healed midline and right upper quadrant scar, the abdomen was nondistended and nontender to palpation, and no masses were noted. Review of previous cross-sectional imaging studies revealed that this mesenteric mass was not present 6 years ago. Thus, malignancy was suspected, with the differential including small bowel carcinoid tumor, metastatic adenocarcinoma, desmoid tumor and gastrointestinal stromal tumor. Positron emission tomography (PET) with fluorodeoxyglucose (FDG) showed a moderately hypermetabolic focus in the periumbilical anterior abdomen with no other abnormal hypermetabolic foci (Figure 2). An octreotide scan was negative. A percutaneous CT guided needle biopsy was obtained and pathology revealed a spindle cell lesion with an inflammatory background and focal palisading necrosis; a spindle cell neoplasm could not be ruled out. Immunohistochemical stain for CD117 (C-kit receptor) was negative.

Given a nondiagnostic core needle biopsy but suggestive of malignancy, along with symptoms of intermittent obstruction, an exploratory laparotomy was undertaken. At exploration, the patient was noted to have extensive thickening and fibrosis of the entire small bowel mesentery along with centimeter sized, firm nodules throughout the mesentery. One nodule measuring approximately 2 × 2 cm was causing severe narrowing of the small bowel. This nodule along with 10 cm of the involved small bowel were resected and sent to pathology for frozen section analysis, which revealed acute and chronic inflammation, extensive necrosis, and foci of partial fibrinoid granulomas. Given the extensive nature of this disease process, no further attempts at resection were made as this disease process was incompatible with complete resection.

**Figure 3:**
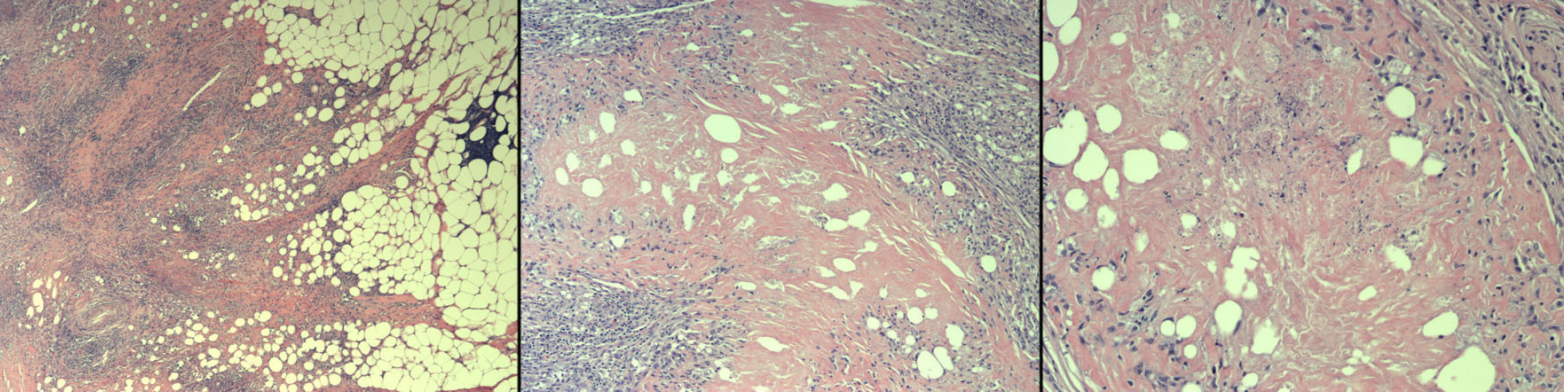
**Permanent pathology sections of the mesenteric mass**. These sections show mesenteric fat necrosis along with inflammation and fibrosis, extending to the subserosal fat of the small intestine. There is no evidence of a neoplasm. (From left to right: 25×, 50×, 100×).

**Figure 4:**
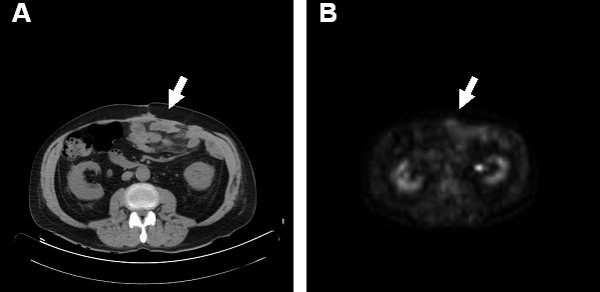
**Imaging studies after RA treatment with etanercept and prednisone**. (A) CT exam of the abdomen demonstrating moderate resolution of the previous mass/lesion shown in Figure 2 Panel A (arrow). (B) FDG-PET transverse image demonstrating decreased tracer uptake in the anterior abdominal lesion after treatment of RA (arrow).

The patient's postoperative course was complicated by a small, superficial, wound infection but was otherwise unremarkable. Final pathology revealed mesenteric fat necrosis along with chronic inflammation and fibrosis, extending to the subserosal fat of the small intestine, and upon consultation with rheumatology, this was deemed to be an unusual presentation of rheumatoid nodules in the mesentery of the small bowel (Figure 3). The patient was re-started on etanercept and low dose prednisone to control his RA. The patient did well without any further complaints of abdominal pain or symptoms of obstruction. He proceeded to gain weight over the next several months and continues to do well. After 4 months of therapy a repeat FDG-PET/CT imaging study was obtained and demonstrated a decrease in the size of the abdominal rheumatoid nodules and no associated FDG-PET activity in the abdomen (FDG-PET negative) (Figure 4).

## Discussion

Rheumatoid arthritis is associated with many extra-articular manifestations, of which rheumatoid nodules are the most common, occurring in approximately 25% of patients with RA[1]. Rheumatoid nodules are more common in Caucasian males and occur more frequently in patients who are RF positive[1]. Rheumatoid nodules occur most commonly at pressure points such as the extensor surfaces of the forearms, fingers, occiput, ischial areas, and the Achilles tendon, but may also occur within internal tissues of the body: central nervous system, heart, pericardium, lungs, pleura, peritoneum, bones, vocal cords, sclera, and the mesentery[1].

From a histological standpoint, rheumatoid nodules are characterized by a central area of necrosis that includes collagen fibrils, fibrin, and proteins[1]. Surrounding this central area are palisading epithelioid cells and chronic inflammatory cells[1]. Fibroblasts are also present within the nodule and produce significant quantities of metalloproteases[7]. Similarly, histology from our case demonstrated extensive mesenteric fat necrosis, chronic inflammation and fibrosis. Immunohistochemical staining of rheumatoid nodules has shown positive staining of epithelioid cells for HLA-DR, CD68, lysozyme, MMP-2, MMP-3, MMP-9 and Ki67[8]. These markers are helpful but may not provide adequate discrimination to rule out a malignancy as many tumors are also positive for MMPs[9].

### Accelerated rheumatoid nodulosis

There have been numerous reports of accelerated rheumatoid nodulosis, defined as a significant increase in the size and number of rheumatoid nodules, secondary to the use of methotrexate[1,10,11]. RF seropositivity seems to be a risk factor for the development of these accelerated nodules, and they usually favor the hands, but can also occur in various other anatomic locations[1]. Most of these accelerated nodules are histologically identical to the classic rheumatoid nodules described earlier[1,11]. In addition to methotrexate, azathioprine has also been associated with this phenomenon of accelerated rheumatoid nodulosis[12].

Our seropositive patient actually had a history of methotrexate use and this may have been the etiological agent responsible for the accelerated rheumatoid nodulosis evident in his small bowel mesentery. This medication was completely discontinued and the patient was instead started on etanercept and low dose prednisone, allowing for good control of his RA and moderate regression of the abdominal rheumatoid nodules.

## Conclusion

Rheumatoid nodules should be included in the differential diagnosis of a patient who presents with an intra-abdominal mass and a history of RA. Special attention should be paid to the medication regimen of a patient with RA, as some of these agents have been shown to exacerbate the growth of rheumatoid nodules.

## Consent

Written informed consent was obtained from the patient for publication of this case report and any accompanying images. A copy of the written consent is available for review by the Editor-in-Chief of this journal.

## Competing interests

The authors declare that they have no competing interests.

## Authors' contributions

ST conceived the idea for the manuscript, conducted a literature search, and drafted the manuscript. JST performed the surgery, critically revised the manuscript, and obtained images used in the manuscript. All authors read and approved the final manuscript.
